# Lactylation of its phosphoprotein interferes with the replication of the spring viremia of carp virus

**DOI:** 10.1128/spectrum.02473-25

**Published:** 2026-07-06

**Authors:** Yanan Song, Zhi Li, Jun Li, Ziyi Li, Le Yuan, Yanyi Wang, Runkun Yan, Fuxiang Lai, Jing Wang, Wuhan Xiao

**Affiliations:** 1 State Key Laboratory of Breeding Biotechnology and Sustainable Aquaculture, Institute of Hydrobiology, Chinese Academy of Sciences; Hubei Hongshan Laboratory 53021 , Wuhan, People’s Republic of China; 2 University of Chinese Academy of Sciences 74519, Beijing, People’s Republic of China; 3 Laboratory for Marine Biology and Biotechnology, Qingdao Marine Science and Technology Center, Qingdao, People’s Republic of China; 4 The Innovation of Seed Design, Chinese Academy of Sciences 53041 , Wuhan, People’s Republic of China; Emory University School of Medicine, Atlanta, Georgia, USA

**Keywords:** SVCV, *ldha*, phosphoprotein, lactylation

## Abstract

The metabolic by-product of glycolysis, lactate, and lactylation-mediated protein modification are involved in various biological processes, including host antiviral responses. The spring viremia of carp virus (SVCV), a rhabdovirus that devastates aquaculture, relies on its multifunctional phosphoprotein (P) for replication and evasion of the host immune system. In this study, we found that SVCV infection increased lactate levels in zebrafish, with lactate modulating SVCV proliferation through lactylation of the viral P protein. *ldha*-null zebrafish exhibited reduced lactate levels and increased susceptibility to SVCV. Conversely, sodium lactate supplementation suppressed viral replication and improved the survival rate of infected zebrafish. Furthermore, we identified lactylation sites in the SVCV P protein. These findings revealed that lactate-mediated lactylation is a host defense mechanism against SVCV and could provide novel strategies for antiviral intervention in aquaculture.

**IMPORTANCE:**

Lactylation is an emerging post-translational modification, yet its presence in viruses has rarely been investigated. This study identified the lactylation modification sites in the spring viremia of carp virus (SVCV) P protein and demonstrated that adding sodium lactate modifies the P protein and reduces SVCV replication. These results suggest that sodium lactate has antiviral properties and plays an important role in combating SVCV infection, while also revealing a novel modification of the SVCV viral protein within the host.

## INTRODUCTION

The spring viremia of carp virus (SVCV), which belongs to the Rhabdoviridae family, poses a significant threat to the global aquaculture industry, due to its high infection and fatality rates in cyprinid fish (1, 2). This negative-sense RNA virus has a conserved genomic architecture (3′-N-P-M-G-L-5′) which encodes five structural proteins: the nucleoprotein (N), the phosphoprotein (P), the matrix protein (M), the glycoprotein (G), and the RNA-dependent RNA polymerase (L). Clinical manifestations of SVCV infection include a characteristic hemorrhagic syndrome with petechiae in cutaneous and visceral tissues and degeneration of the gill lamellae (3). SVCV replication relies heavily on the multifunctional phosphoprotein (P protein), which reportedly acts as a cofactor for viral RNA polymerase (vRNApol) in regulating viral protein transcription (4–7). Additionally, the P protein is reported to modulate the host antiviral response by inhibiting TANK binding kinase 1 (TBK1) (8). Understanding how host factors affect SVCV replication will help to develop approaches for preventing SVCV disease in fish.

In the context of animal metabolism, glycolysis occurs in the cytoplasm under aerobic conditions and results in the breakdown of hexose into pyruvate. The resulting pyruvate then enters the mitochondria, where it is used in the tricarboxylic acid cycle. Under hypoxic conditions, however, pyruvate is converted to lactate through lactic acid fermentation, catalyzed by lactate dehydrogenase (9, 10). In recent years, the pivotal role of lactate in biological processes, particularly the immune response, has received more attention (11).

Post-translational modifications (PTMs) of viral proteins are being increasingly recognized as the key regulators of viral pathogenicity. For instance, threonine phosphorylation at position 160 of the snakehead vesiculovirus phosphoprotein has been shown to promote viral replication (12), Tripartite motif containing 7 (TRIM7) has been found to ubiquitinate enterovirus 2BC and degrade viral proteins via the proteasome pathway (13); and the acetylation of the viral nonstructural protein NS3 helicase by KAT5γ has been demonstrated to promote flavivirus replication (14). However, the role of lactylation, a novel lysine-targeted PTM driven by lactate, in regulating viral protein function remains largely unexplored.

Recent studies have revealed that lactylation dynamically regulates cellular processes, including chromatin remodeling and metabolic signaling (15, 16), by linking glycolysis to protein function. Intriguingly, many viruses, including SVCV, elicit various pathophysiological responses in host cells to favor glycolysis. This raises the possibility that increased lactate content in the microcycle during infection may promote the lactylation of viral proteins, thereby affecting their activity. The SVCV P protein, with its intrinsically disordered regions and lysine-rich domains (17), is a plausible substrate for lactylation. This study, therefore, investigated the functional implications of P protein lactylation during SVCV proliferation to gain new insights into how viruses use host metabolic pathways for post-translational modifications. The findings of this study could inform the development of novel antiviral strategies targeting lactylation modifications in aquaculture pathogens.

## RESULTS

### *ldha*-null zebrafish larvae are susceptible to SVCV infection

**Fig 1:**
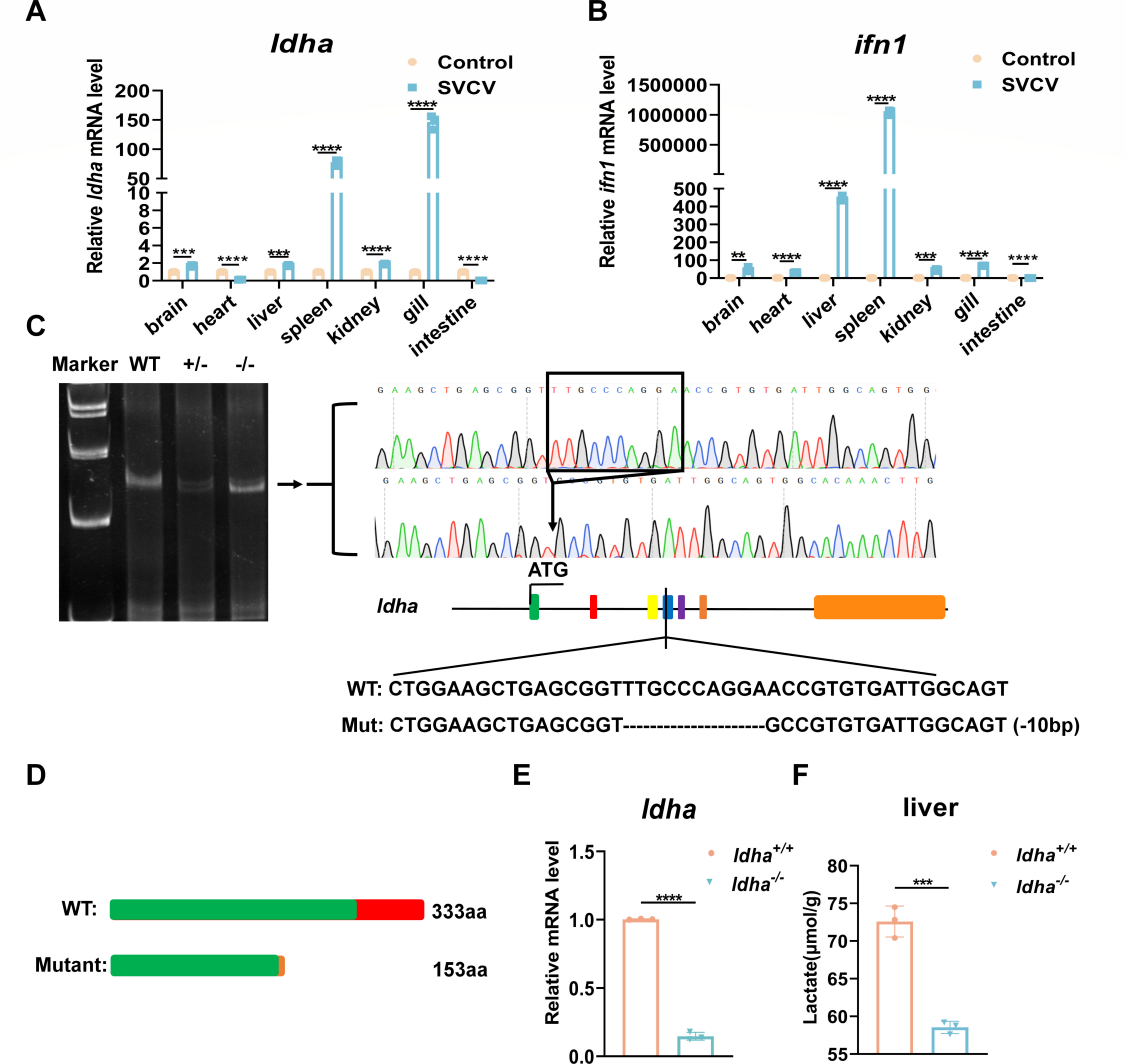
Generation of *ldha*-null zebrafish by CRISPR/Cas9. (**A and B**) *ldha* (**A**) or *ifn1* (**B**) was induced in zebrafish tissues (3 months post-fertilization [mpf]) by SVCV infection. (**C**) CRISPR/Cas9-mediated disruption of the zebrafish *ldha* gene was validated using a heteroduplex mobility assay (HMA). The targeting site and mutant allele (*ldha*^ihbsyn02/ihbsyn02^) are depicted schematically. (**D**) The wild-type (WT) ldha protein in WT zebrafish and the predicted truncated peptide in the mutant zebrafish. (**E**) Expression of *ldha* in the zebrafish larvae (4 days post-fertilization [dpf]) mutants and their WT siblings. (**F**) Lactic acid level in the livers of mutants and their WT siblings. The data presented are the means ± SEM, based on one representative experiment performed in triplicate. *P < 0.05; **P < 0.01; ***P < 0.001; ****P < 0.0001.

Given that LDHA plays a role in regulating lactate production during metabolism in animals, and that lactate has been reported to be involved in antiviral immunity (11), we sought to investigate the role of fish *ldha* and lactate in the antiviral response. First, we examined the expression of *ldha* in various tissues of SVCV-infected zebrafish using quantitative real-time PCR (qRT-PCR). The results revealed that *ldha* could be significantly induced by SVCV infection, in parallel with the expression pattern of the canonical antiviral gene *ifn1* (Fig. 1A and B). This coordinated induction of *ldha* suggested a potential link between ldha/lactate and the antiviral response in fish.

To further investigate the role of fish *ldha* in the antiviral responses *in vivo*, we generated *ldha*-null zebrafish using the CRISPR/Cas9 technology. We created a mutant line with a 10-nucleotide deletion in exon 4 of the *ldha* (*ldha*^ihbsyn02/ihbsyn02^) (Fig. 1C and D). qRT-PCR results confirmed the efficient disruption of *ldha* transcription (Fig. 1E). By crossing *ldha*^+/–^ (♀) and *ldha*^+/–^ (♂), the offspring with *ldha*^+/+^, *ldha*^+/–^, and *ldha*^−/–^ genetic backgrounds were obtained at a Mendelian ratio (1:2:1). No obvious defects in the growth rate or reproductive capability were detected in *ldha*^−/–^ zebrafish under normal conditions. To verify the effect of *ldha* on lactate production, we performed a lactate assay, revealing that the *ldha* knockout significantly reduced the lactate content in zebrafish livers (Fig. 1F).

**Fig 2:**
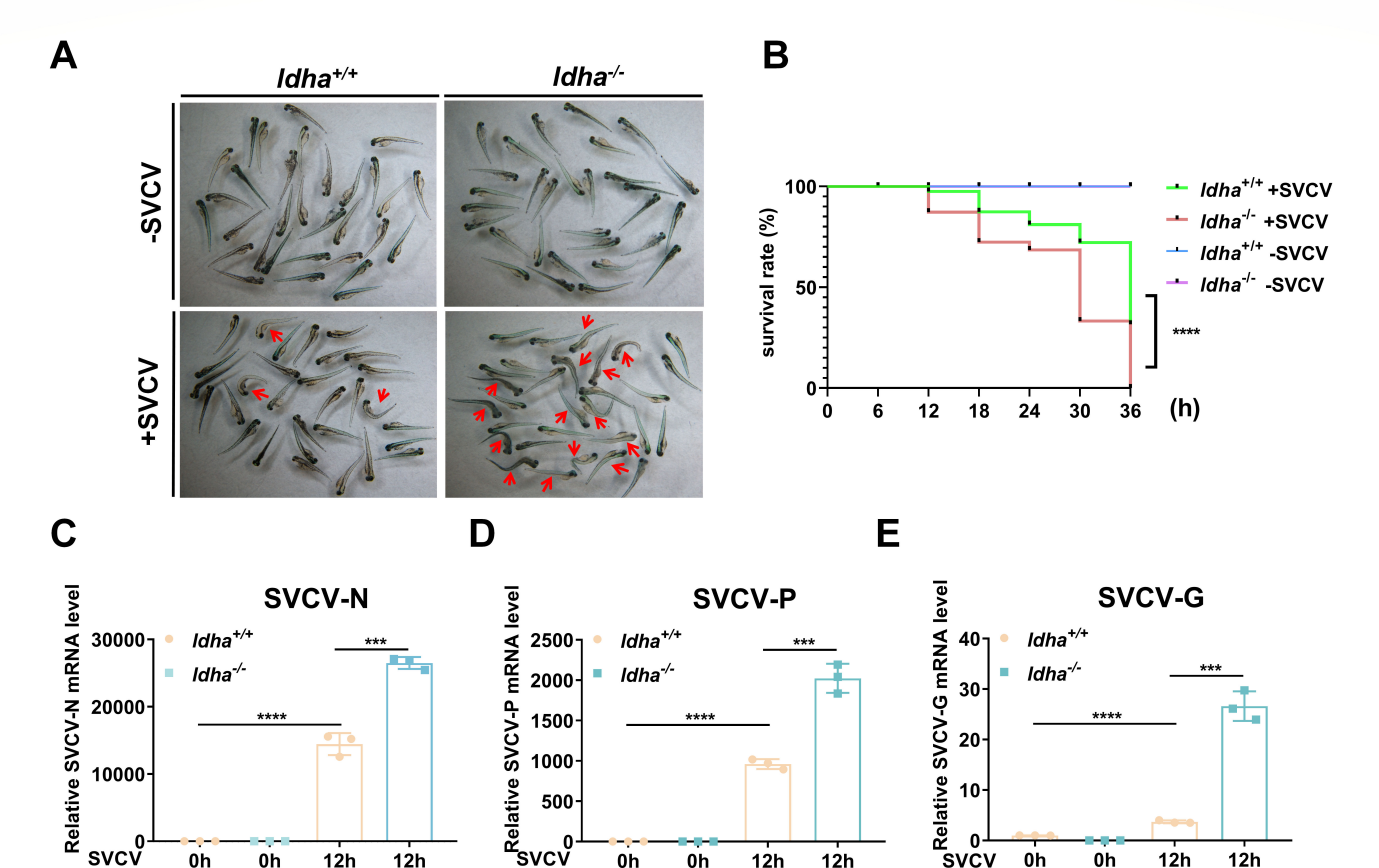
*ldha*-null zebrafish larvae are susceptible to SVCV infection. (**A**) Images of *ldha*^+/+^ and *ldha*^−/−^ larvae (4 dpf) infected (+; 12 h) or uninfected (−) with SVCV. Red arrows indicate dead larvae, characterized by immobility, the absence of circulation, and a degenerated body. (**B**) Survival curves for *ldha*^+/+^ (*n* = 96) and *ldha*^−/−^ (*n* = 96) larvae after infection (36 h) or no infection with SVCV (~2.5 × 10^7^ 50% tissue culture-infective dose [TCID_50_]/mL). (**C–E**) qRT-PCR analysis of N (**C**), P (**D**), and G (**E**) protein mRNA of SVCV in *ldha*^+/+^ and *ldha*^−/−^ larvae 12 hpi. The data presented are the means ± SEM, based on one representative experiment performed in triplicate. *P < 0.05; **P < 0.01; ***P < 0.001; ****P < 0.0001.

Next, we challenged zebrafish larvae with SVCV. Twelve hours post-infection (hpi), a markedly greater proportion of SVCV-infected *ldha*^–/–^ larvae (4 days post-fertilization, dpf) were dead compared to the wild-type (WT) controls. This was evidenced by immobility, an absence of circulation and body degeneration (Fig. 2A). Survival curves revealed a significantly lower survival rate in the *ldha*^–/–^ larvae, with mortality occurring earlier and more rapidly than in their WT siblings (Fig. 2B). Consistent with the survival results, viral replication was substantially increased in the *ldha*^–/–^ larvae, as demonstrated by markedly elevated mRNA levels of the SVCV nucleocapsid (N), phosphoprotein (P), and glycoprotein (G) genes at 12 hpi (Fig. 2C through E).

These data suggest that disruption of *ldha* promotes SVCV replication in zebrafish larvae.

### *ldha*-null zebrafish adults are susceptible to SVCV infection

**Fig 3:**
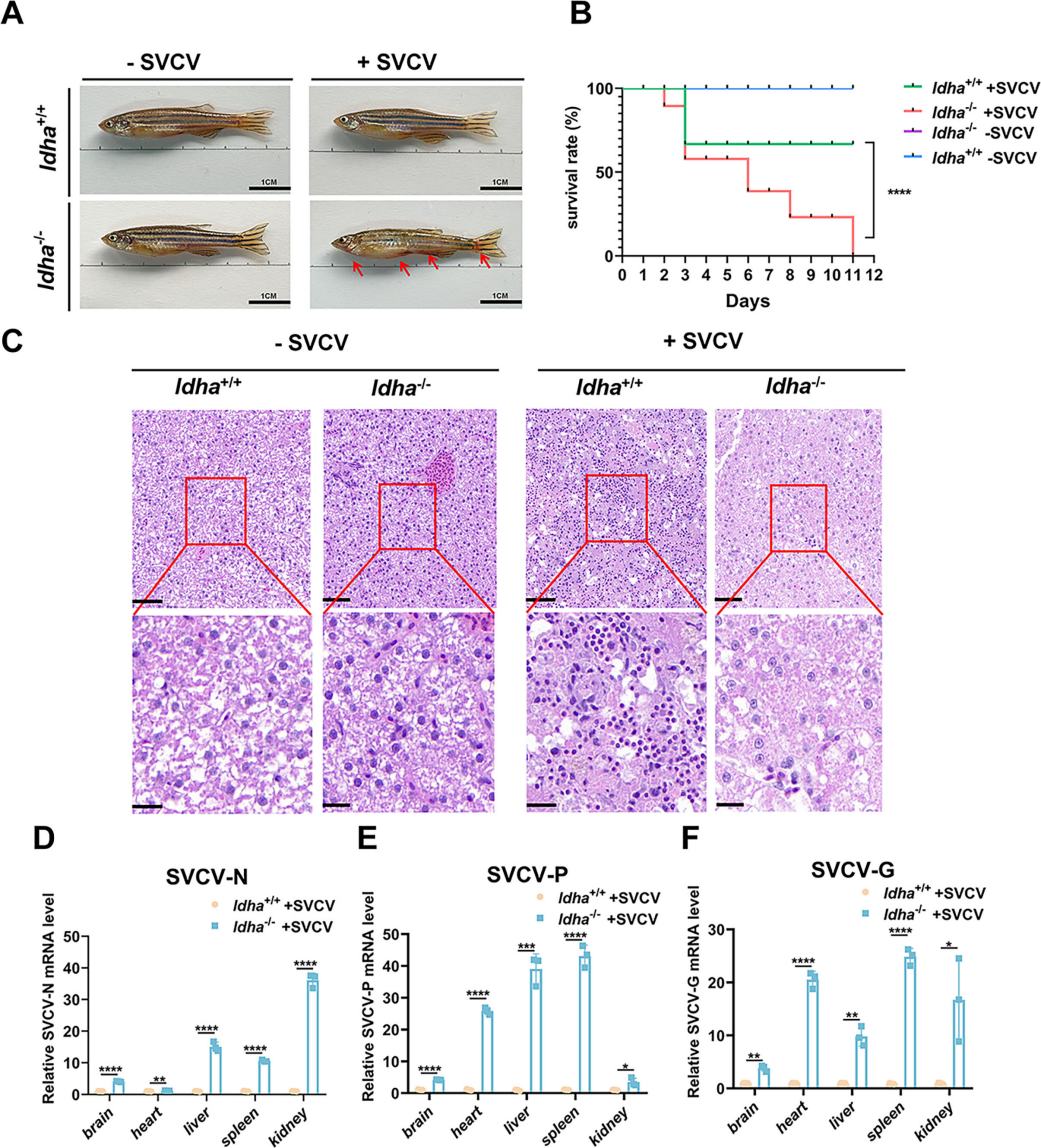
*ldha*-null adult zebrafish are susceptible to SVCV infection. (**A**) The *ldha*^−/−^ zebrafish (3 mpf) displayed enhanced abdominal hemorrhaging compared to the WT controls following the i.p. injection of 10 μL SVCV (~2.5 × 10⁷ TCID₅₀/mL) versus the cell culture medium. The hemorrhagic regions are indicated by red arrows. (**B**) Survival curves for SVCV-infected *ldha*^−/−^ zebrafish (*n* = 20) and their wild-type siblings (*n* = 20). (**C**) Hepatic histopathology revealed greater necrosis and cytoplasmic vacuolization in SVCV-infected *ldha*^−/−^ zebrafish than in their WT siblings. (**D–F**) qRT-PCR analysis of N (**D**), P (**E**), and G (**F**) protein mRNA of SVCV in *ldha*^+/+^ and *ldha*^−/−^ adult zebrafish (3 mpf) 48 hpi. The data presented are the means ± SEM, based on one representative experiment performed in triplicate. *P < 0.05; **P < 0.01; ***P < 0.001; ****P < 0.0001.

We then challenged *ldha*^+/+^ and *ldha*^–/–^ zebrafish adults (3 months post-fertilization, mpf) with SVCV. The intraperitoneal (i.p.) injection of SVCV induced pronounced abdominal hemorrhage in the *ldha*^–/–^ zebrafish, a phenotype rarely observed in the infected WT controls (Fig. 3A). Survival curves revealed more mortality in the *ldha*^−/−^ adults than WT counterparts (Fig. 3B). Histopathological analysis of the liver revealed that the *ldha*^−/−^ zebrafish presented more severe pathologies, including extensive necrosis and cytoplasmic vacuolation, than the WT zebrafish (Fig. 3C). qRT-PCR revealed that the expressions of the SVCV N, P, and G genes in the brain, heart, liver, spleen, and kidney of the *ldha*^–/–^ zebrafish were higher than those in the WT zebrafish tissues (Fig. 3D through F).

Taken together, these data highlight that *ldha* also limits viral proliferation in zebrafish adults.

### The addition of sodium lactate to living water protects against SVCV in zebrafish larvae

**Fig 4:**
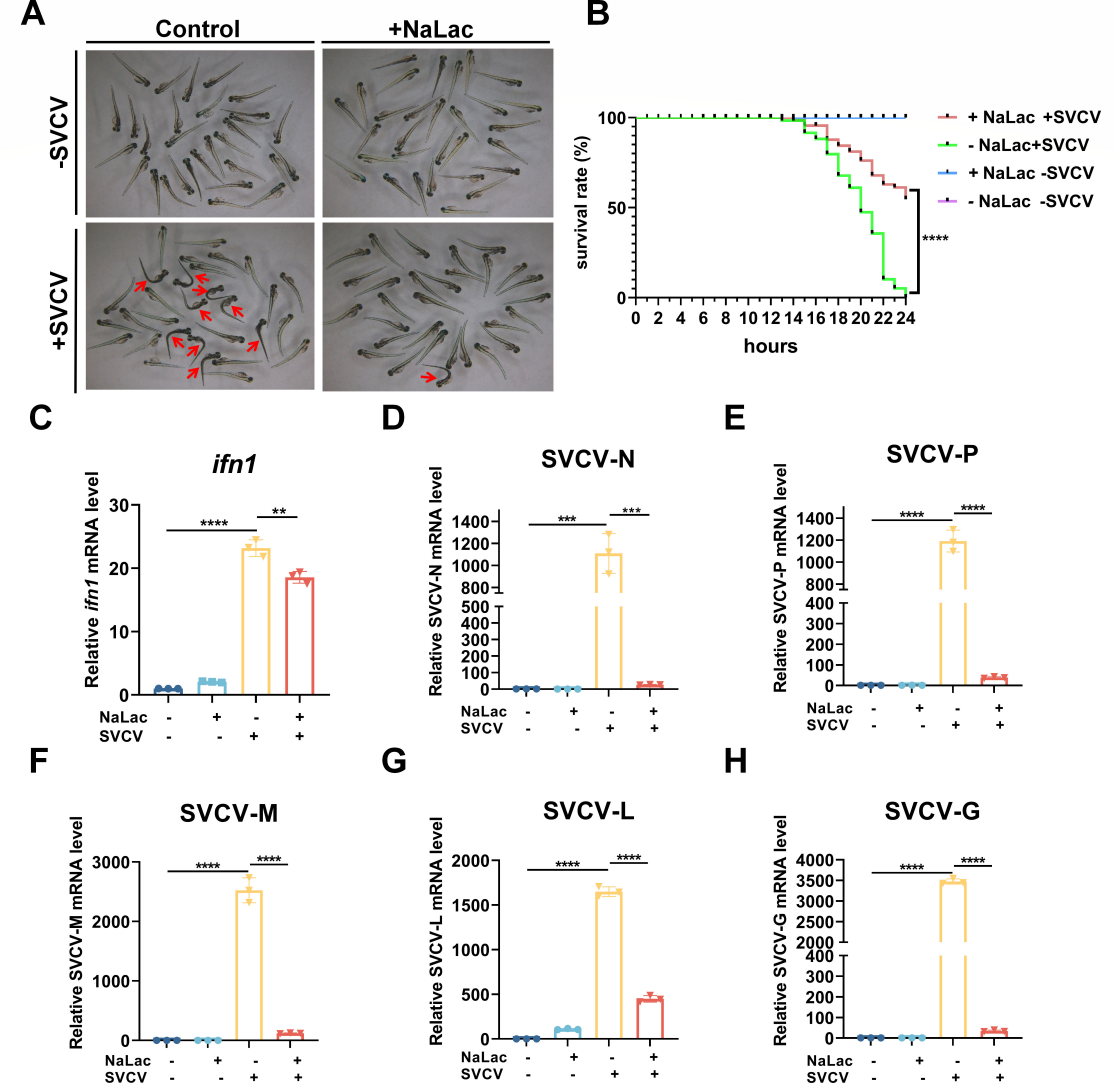
The addition of sodium lactate to living water protects against SVCV in zebrafish larvae. (**A**) Images of zebrafish larvae (4 dpf) treated with sodium lactate (5 mM) or sea salt (5 mM) control after being infected (12 h) or not infected with SVCV (~2.5 × 10^7^ TCID_50_/mL). Red arrows indicate the deceased larvae, characterized by motility cessation, circulatory arrest, and body degeneration. (**B**) Survival curves for zebrafish larvae treated with sodium lactate (5 mM) (*n* = 96) or sea salt (5 mM) control after infection or no infection with SVCV (~2.5 × 10^7^ TCID_50_/mL), monitored hourly for up to 24 h after SVCV exposure. (**C**) qRT-PCR analysis of *ifn1* mRNA in zebrafish larvae treated with sodium lactate (5 mM) or sea salt (5 mM) control after infection (12 h) with or without SVCV (~2.5 × 10^7^ TCID_50_/mL). (**D–H**) qRT-PCR analysis of N (**D**), P (**E**), M (**F**), L (**G**), and G (**H**) protein mRNA in zebrafish larvae treated with sodium lactate (5 mM) or sea salt control (5 mM) control after infection (12 h) with or without SVCV (~2.5 × 10^7^ TCID_50_/mL). The data presented are the means ± SEM, based on one representative experiment performed in triplicate. *P < 0.05; **P < 0.01; ***P < 0.001; ****P < 0.0001.

Given that *ldha* mediates lactate production, we sought to directly investigate the role of lactate in SVCV proliferation. As zebrafish absorb compounds from the aquatic environment (18), we added sodium lactate to the living water of zebrafish larvae prior to SVCV challenge. After 12 hpi, the larvae treated with sodium lactate exhibited a substantially lesser mortality compared to the control counterparts, as evidenced by the reduced numbers of deceased individuals displaying motility cessation, circulatory arrest, and body degeneration (Fig. 4A). Survival analysis confirmed a significant improvement in survival rates among the sodium lactate-treated larvae following SVCV infection (Fig. 4B). Consistent with the phenotypic observations, typical antiviral gene *ifn1* was downregulated and viral replication was markedly suppressed in the sodium lactate-treated larvae, with significantly reduced mRNA levels of the SVCV N, P, M, L, and G genes at 12 hpi (Fig. 4C through H).

These data suggest that the addition of sodium lactate to living water protects against SVCV in zebrafish larvae.

### Dietary supplementation with sodium lactate provides protection against SVCV in adult zebrafish

**Fig 5:**
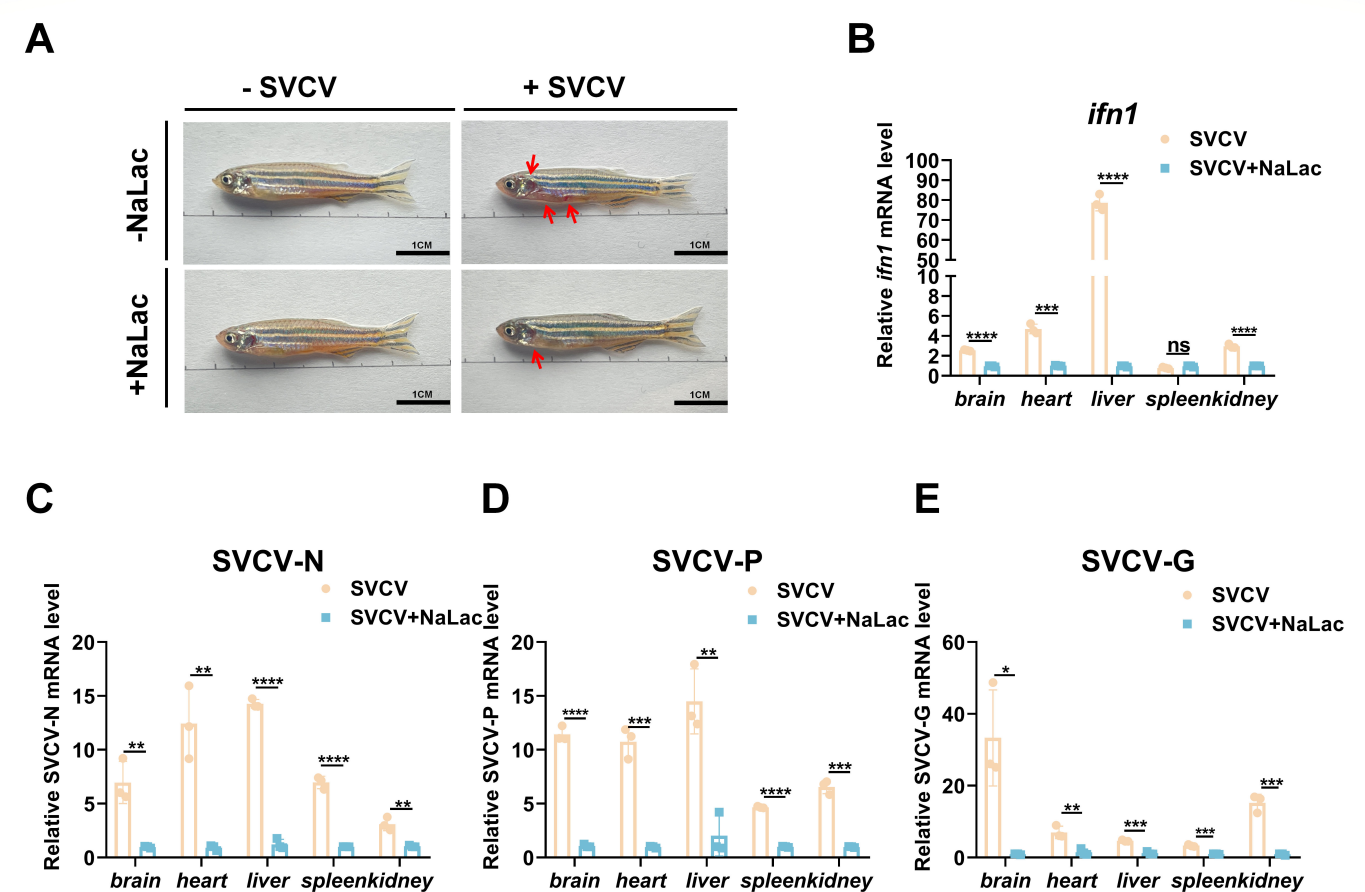
Dietary supplementation with sodium lactate provides protection against SVCV in adult zebrafish. (**A**) Images of adult zebrafish (3 mpf) fed either sodium lactate-supplemented food or control food, either infected or uninfected with SVCV (~2.5 × 10^7^ TCID_50_/mL). The hemorrhagic regions are marked by red arrows. (**B**) qRT-PCR analysis of *ifn1* mRNA in adult zebrafish (3 mpf) fed sodium lactate-supplemented food or control food, either infected or uninfected with SVCV (~2.5 × 10^7^ TCID_50_/mL), for 48 h. (**C and D**) qRT-PCR analysis of N (**C**), P (**D**), and G (**E**) SVCV protein mRNA in multiple organs (brain, heart, liver, spleen, and kidney) of adult zebrafish (3 mpf) fed sodium lactate-supplemented food or control food and infected or uninfected with SVCV (~2.5 × 10^7^ TCID_50_/mL) for 48 h. The data presented are the means ± SEM, based on one representative experiment performed in triplicate. *P < 0.05; **P < 0.01; ***P < 0.001; ****P < 0.0001.

In the aquatic animal feed industry, numerous organic compounds are selected as additives to enhance feed efficiency and improve physiological and immune responses in fish (19, 20). We also treated adult zebrafish (3 mpf) with a sodium lactate-supplemented diet. Without a sodium lactate-supplemented diet, zebrafish displayed exacerbated pathological manifestations post-SVCV infection, including pronounced abdominal hemorrhaging, which was rarely observed in zebrafish with a sodium lactate-supplemented diet (Fig. 5A). To ascertain the mechanism by which sodium lactate influences the capacity of zebrafish to protect against SVCV infection, we examined the expression of the antiviral gene *ifn1*. We found that SVCV induced higher expression of *ifn1* and the SVCV N, P, and G genes in zebrafish without a sodium lactate-supplemented diet than in those with a sodium lactate-supplemented diet (Fig. 5B through E).

These data suggest that dietary supplementation with sodium lactate protects against SVCV in adult zebrafish.

### Sodium lactate suppresses SVCV proliferation in EPC cells

**Fig 6:**
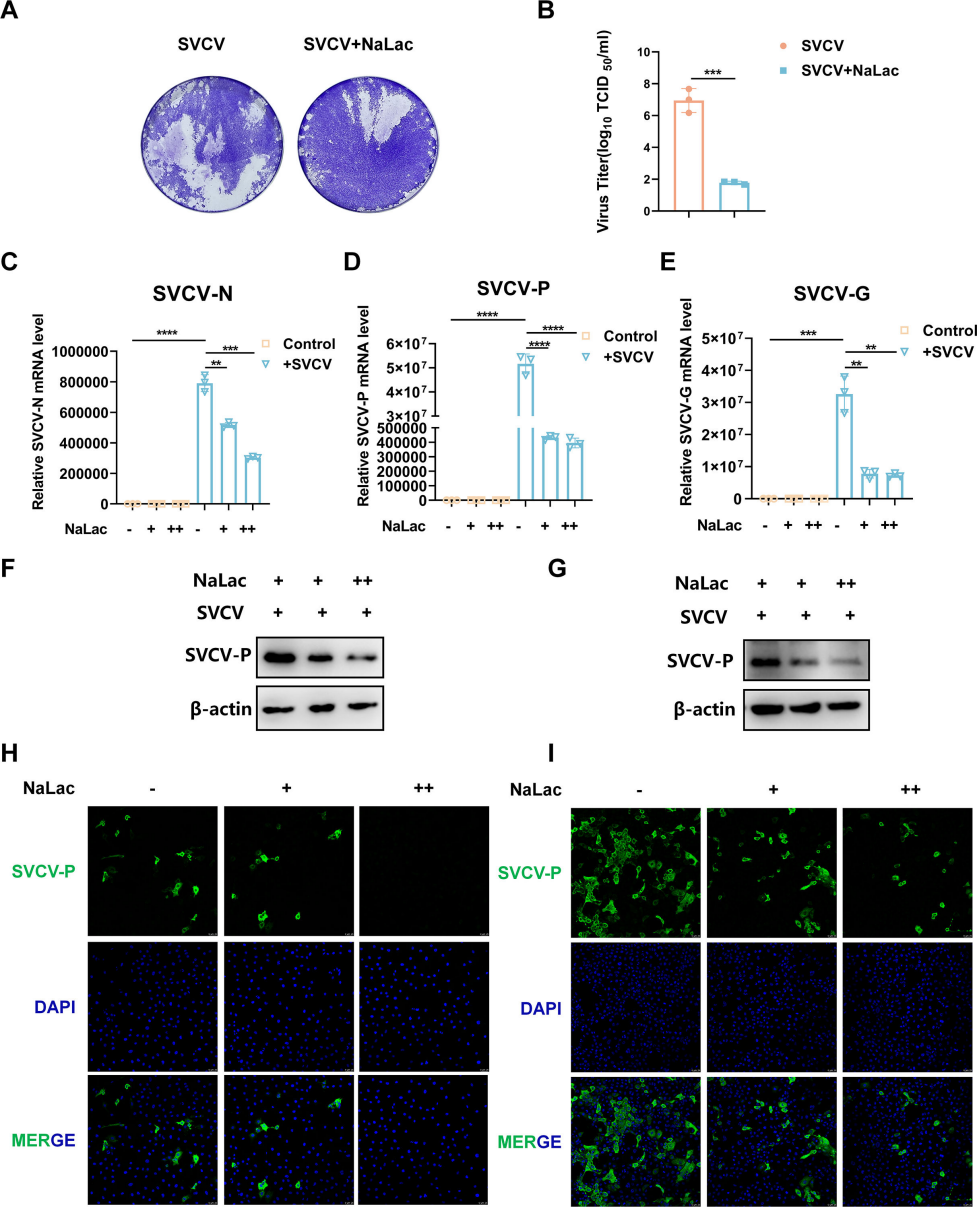
The supplementation of sodium lactate suppresses SVCV proliferation in EPC and ZFL cells. (**A**) Sodium lactate treatment improved the survival of SVCV-infected EPC cells. The cells were pretreated with sodium lactate (5 mM) or phosphate-buffered saline (PBS) for 12 h prior to infection with SVCV at a multiplicity of infection (MOI) of 1. After 48 h of infection, the cells were fixed with 4% paraformaldehyde and then stained with 1% crystal violet to assess viability. (**B**) Reduction of viral titers in infected EPC cells upon treatment with sodium lactate. Supernatants from the SVCV-infected cells were collected and used to quantify the viral titer using a plaque assay. The results are representative of three independent experiments. (**C–E**) The addition of sodium lactate attenuated the SVCV gene expression in the infected EPC cells. At 24 h post-infection with SVCV and following pretreatment with 5 mM sodium lactate/PBS, total RNA was extracted for qRT-PCR analysis to determine the mRNA of the SVCV N (**C**), P (**D**), and G (**E**) proteins. (**F–I**) Western blot and immunofluorescence analysis of the antiviral effects of sodium lactate in ZFL and EPC cells. ZFL (**F**) and EPC (**G**) cells were pretreated with various concentrations of sodium lactate for 12 h, then infected with SVCV for a further 24 h. Whole-cell lysates were immunoblotted with an anti-SVCV-P antibody. Immunofluorescence analysis and confocal microscopy were performed on ZFL (**H**) and EPC (**I**) cells cultured on glass-bottom dishes. Following identical pretreatment and infection protocols, the cells were fixed and immunostained using an anti-SVCV-P antibody. The data presented are the means ± SEM, based on one representative experiment performed in triplicate. *P < 0.05; **P < 0.01; ***P < 0.001; ****P < 0.0001.

We then validated the antiviral ability of sodium lactate in epithelioma papulosum cyprini (EPC. Pretreating EPC cells with sodium lactate reduced the cytopathic effect (CPE), indicating a higher survival rate of cells after SVCV infection (Fig. 6A). Compared to the controls, the viral titers in the cell culture supernatant from the sodium lactate-treated cells were markedly lower (Fig. 6B). Consistent with these findings, expression of the SVCV N, P, and G genes was lower in sodium lactate-pretreated cells (Fig. 6C through E). Immunoblotting and immunofluorescence analyses corroborated these antiviral effects, revealing reduced SVCV-P protein levels in both zebrafish liver (ZFL) and EPC cell lines following sodium lactate pre-treatment (Fig. 6F through I). These data suggest that lactate is a potent inhibitor of SVCV replication.

### SVCV infection increases the level of lactic acid in cells and enhances protein lactylation

To determine the mechanism underlying the inhibition of SVCV proliferation by sodium lactate treatment, we examined whether sodium lactate inhibits viral proliferation by directly affecting viral proteins. Immunoblotting was used to determine the effect of sodium lactate on the stability of the N, P, and G proteins of SVCV. As shown in Fig. S1, gradient addition of sodium lactate did not affect the stability of the overexpressed N, P, and G proteins in human embryonic kidney (HEK) 293T cells. These results indicated that sodium lactate did not interfere with the expression of the SVCV protein. Lactate reportedly targets MAVS to negatively regulate the RLR pathway in mammals (21). Therefore, we then checked the immunomodulatory effects of lactate on RLR pathway activation. In the VSV-challenged THP-1 cells, both lactate and sodium lactate markedly reduced the virus-induced expressions of *IFNB1*, *CXCL10*, and *ISG15* (Fig. S2A through C). A parallel suppression pattern emerged in the poly(I:C)-stimulated HEK293T cells, with lactate treatment resulting in attenuating *IFNB1, CXCL10*, and *ISG15* expression (Fig. S2D through F). These data not only supported the previous report (21), but also indicated that the analysis method employed in this study was reliable. However, neither sodium lactate nor lactate altered poly(I:C)-triggered transcriptional upregulation of *ifn* or *viperin* in fish EPC cells (Fig. S3A and B), in contrast to the effect exhibited in mammalian cells. Collectively, these findings suggest that the modulation of antiviral signaling by lactate derivatives was host cell type-specific, which highlights the fundamental differences in the metabolic-immune crosstalk between mammalian and fish cell lineages.

**Fig 7:**
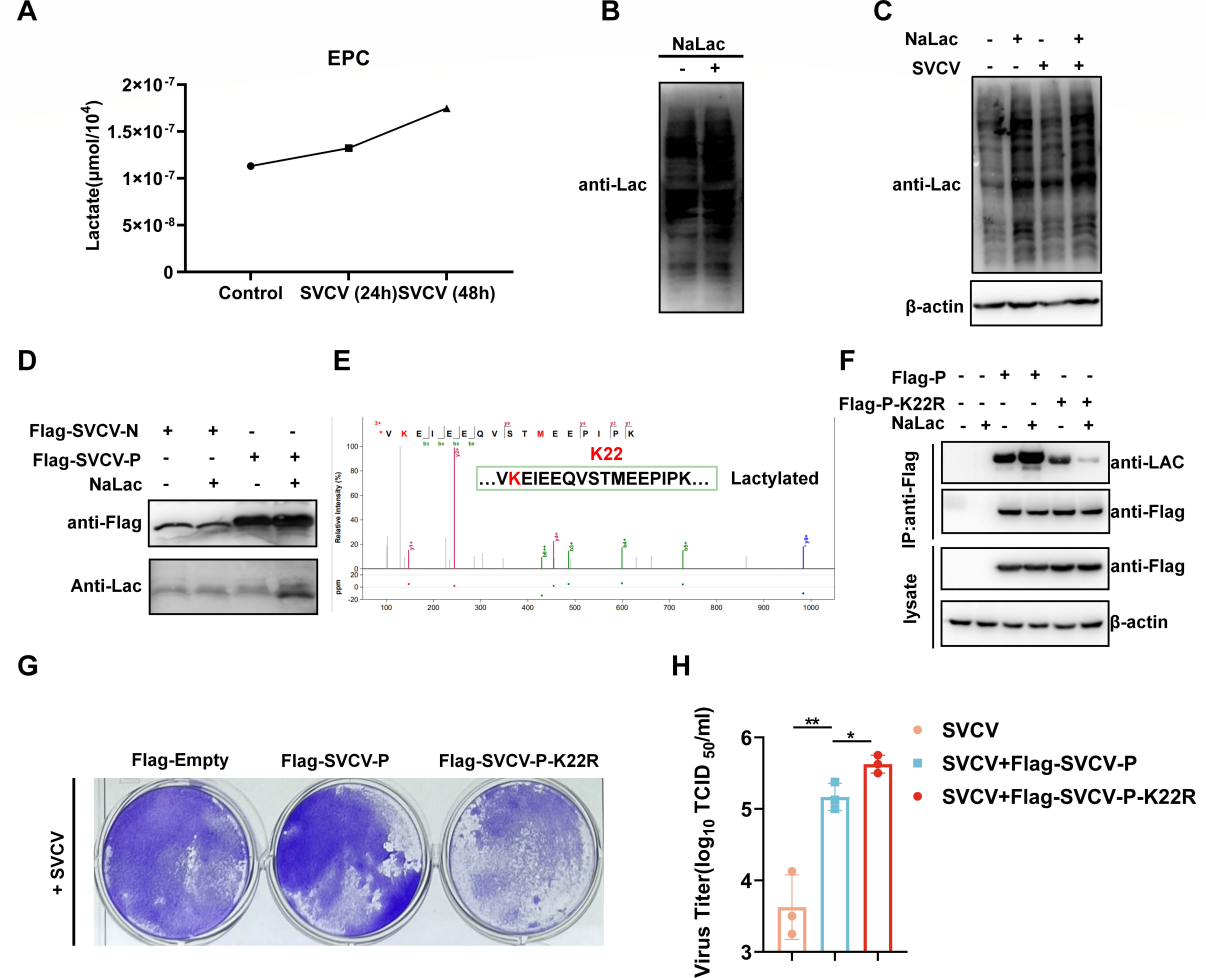
SVCV infection increases the level of lactic acid in cells and enhances protein lactylation, including that at lysine 22 of the SVCV P protein. (**A**) Lactate quantification in EPC cells after SVCV infection. EPC cells were cultured in 6-well plates and infected with SVCV. The lysates were collected at 24 h and 48 h post-infection and analyzed using lactate assay kits. Uninfected cells served as a control. (**B**) Western blot analysis revealed increased lactylation in EPC cells following sodium lactate treatment. EPC cells were plated in 10 cm dishes, incubated with sodium lactate for 24 h, and then the cell lysates were immunoblotted with an anti-lactylation antibody. (**C**) Sodium lactate treatment and SVCV infection synergistically increased protein lactylation in the cells. EPC cells in 10 cm dishes were treated with sodium lactate, SVCV, or both for 24 h, after which the lysates were analyzed for protein lactylation. (**D**) Sodium lactate treatment increased the lactylation of the overexpressed SVCV P protein in HEK293T cells. Cells transfected with Flag-P or Flag-N (negative control) were treated or left untreated with sodium lactate for 24 h, after which the cell lysates were analyzed for protein lactylation. (**E**) Mass spectrometry analysis identified lysine 22 (K22) of SVCV P protein as a lactylation site. HEK293T cells transfected with Flag-P for 24 h were subjected to mass spectrometry analysis. (**F**) Sodium lactate treatment increased the lactylation of the wild-type P protein of SVCV, but not the mutant, P-K22R. HEK293T cells were either treated or left untreated with sodium lactate following transfection with Flag-P or Flag-P-K22R. Protein lactylation was detected by immunoblotting. (**G and H**) Overexpression of SVCV-P reduced the viability of SVCV-infected EPC cells, and Flag-P-K22R exacerbated this effect. EPC cells were transfected with Flag-SVCV-P, Flag-SVCV-P-K22R, or an empty vector control. After 24 h, the cells were infected with SVCV (MOI of 1) for 48 h, then fixed with formaldehyde and stained with crystal violet. The viral titers of the culture supernatants were quantified using a plaque assay. The data presented are the means ± SEM, based on one representative experiment performed in triplicate. *P < 0.05; **P < 0.01; ***P < 0.001; ****P < 0.0001.

Aerobic glycolysis is a common marker of viral infection (22). Lactate quantification experiments conducted in this study demonstrated that SVCV infection induced the accumulation of lactate in EPC cells in a time-dependent manner (Fig. 7A). Previous studies have shown that lactate induces lactylation through the covalent modification of target proteins (23), and that supplementing with exogenous lactate substantially increases protein lactylation levels (24). In this study, sodium lactate treatment was found to further amplify global protein lactylation in EPC cells, as evidenced by immunoblotting (Fig. 7B). Various viruses, including white spot syndrome virus and human cytomegalovirus, have been reported to increase host lactylation levels by modulating host glycolysis following infection (25, 26). Figure 7C shows that SVCV infection combined with sodium lactate exposure synergistically increases lactylation levels. To further investigate the lactylation of SVCV proteins, we performed a mass spectrometry analysis of SVCV-N and P proteins after co-immunoprecipitation using an anti-Flag antibody. This revealed some lactylation modification sites in the SVCV-N and P proteins (Fig. S4A). Further immunoblotting revealed lactylation modification in the P protein (Fig. 7D). Lysine 22 (K22) is a reliable lactylation site on the SVCV-P protein (Fig. 7E; Fig. S4B through F). Overexpression of Flag-P in HEK293T cells confirmed that sodium lactate selectively enhances K22 lactylation, whereas the K22R mutation eliminates this modification (Fig. 7E and F). Overexpression of wild-type SVCV-P reduced the viability of infected EPC cells, whereas the K22R mutation exacerbated cell death and viral titers (Fig. 7G and H).

Taken together, these data suggest that SVCV infection increases the level of lactylation in the host and lactylation of the P protein inhibits viral replication.

## DISCUSSION

In recent years, studies have focused on the pivotal role of lactate, a glucose metabolite, in regulating various biological processes. This includes modulating fat metabolism by suppressing the activity of carnitine palmitoyltransferase 2 (27), lactate’s anti-inflammatory effects (28), and the dysregulation of lactate metabolism and signaling associated with cancer development (29–31). The interplay between the host metabolism and antiviral defense has emerged as a critical area of research in virology (32–34). This study demonstrated that *ldha* deficiency in zebrafish negatively affects lactate production, resulting in increased SVCV replication.

Lactate dehydrogenase is a pivotal enzyme in the aerobic glycolysis process and catalyzes the final step of glycolysis. LDH is a homo- or heterotetramer consisting of LDH-A and LDH-B subunits. The *ldha* gene encodes lactate dehydrogenase A, a subunit of lactate dehydrogenase that catalyzes the reduction of pyruvate to lactate during glycolysis, playing a key role in lactate production in animals (35, 36). In the present study, we found that reduced lactate in zebrafish due to *ldha* knockout promotes SVCV proliferation.

SVCV belongs to the Rhabdoviridae family and is characterized by the presence of five major structural proteins, namely, nucleoprotein (N), phosphoprotein (P), matrix protein (M), glycoprotein (G), and RNA polymerase (L). This study identified a previously unreported lactylation site on lysine in the P protein, suggesting that P proteins from other members of this family of viruses, including the rabies virus and vesicular stomatitis viruses, may also be capable of regulating proliferation in response to lactate metabolism. This finding could inform further research into these viruses, particularly with regard to developing effective prevention methods and robust control strategies.

Lactylation has been shown to play an important role in host–pathogen interactions. Recent studies have focused on viral infections that alter host metabolism and consequently affect host immunity (25, 37). Furthermore, some studies have reported the presence of lactylation modification sites on pathogens (26, 38, 39). This study identified a previously unidentified lactate modification, the lysine site, in the P protein. Additionally, it was revealed that this site is associated with viral proliferation. As P proteins play important roles in SVCV transcription and replication (40–42), this study verified that viral proliferation is inhibited when P proteins are lactylated, but not when the lactylation modification site of P proteins is mutated. This suggests that SVCV proliferation is inhibited by lactate through the lactylation of P proteins. These findings indicate that the host employs lactate metabolism as an effective defense mechanism against viral infections.

Significant losses are caused to the aquaculture industry by SVC disease. However, there is still no operational strategy for controlling the disease. The present study found that dietary lactate supplementation could be a sustainable way to reduce the impact of SVCV outbreaks in aquaculture. This study has provided novel ideas for preventing and controlling SVCV in fish farming.

## MATERIALS AND METHODS

### Cell culture and transfection

HEK293T cells were cultured in DMEM (Biological Industries) supplemented with 10% fetal bovine serum (FBS). THP-1 cells were propagated in RPMI 1640 (Biological Industries), supplemented with 10% FBS. ZFL cells were cultured in a 1:1 mixture of DMEM/F12 (Biological Industries), while EPC cells were grown in Medium 199 (Biological Industries), both supplemented with 10% FBS. The incubation conditions were 37°C for the mammalian cells (HEK293T and THP-1) and 28°C for the ZFL and EPC cells. All cultures were kept in a humidified atmosphere with 5% CO_2_. Prior to experimentation, all cell lines underwent mycoplasma screening using PCR-based detection. Neofect DNA transfection reagent (Neofect) was used for cell transfection.

### Zebrafish

Zebrafish (*Danio rerio*) strain AB were raised, maintained, and staged according to standard protocols. We used CRISPR/Cas9 to knockout the *ldha* gene in zebrafish. First, the *ldha* sgRNA was designed using the CRISPR Design Tool (http://crispr.mit.edu). The Zebrafish Codon Optimized Cas9 plasmid was digested with XbaI, purified, and transcribed using the T7 mMessage mMachine Kit (Ambion). We used a PUC9 gRNA vector to amplify the *ldha* sgRNA template. The primers used to amplify the *ldha* sgRNA were as follows: forward primer (5′-GTAATACGACTCACTATAGGAAGCTGAGCGGTTTGCCCGTTTTAGAGCTAGAAATAGC-3′); and the reverse primer (5′-AAAAGCACCGACTCGGTGCC-3′). The sgRNA was synthesized using the Transcript Aid T7 High Yield Transcription Kit (Fermentas). We injected zebrafish embryos at the one-cell stage (generated as described above) with 1 ng of Cas9 RNA and 0.15 ng of sgRNA per embryo. The mutations were initially detected using heteroduplex mobility assay (HMA), as previously described (43). If the HMA results were positive, the remaining embryos were raised to adulthood as the F0 generation and then backcrossed with WT zebrafish (strain AB) to generate the F1 generation. The F1 generation was genotyped using HMAs. Genotype was confirmed by sequencing the target sites. Heterozygous F1s were backcrossed with WT zebrafish (strain AB; disallowing offspring-parent matings) to generate the F2 generation. F2 adults carrying the target mutation were intercrossed to generate F3 offspring. The F3 generation contained WT (+/+), heterozygous (+/−), and homozygous (−/−) individuals. The primers used to identify the mutants were as follows: forward primer (5′-TACAGCCCCAACTGCATCCTT-3′); reverse primer (5′-ATCAGTGCTCACCGCTGGAGTCT-3′). The novel mutants were named in accordance with zebrafish nomenclature guidelines, *ldha*^ihbsyn02/ihbsyn02^ (https://zfin.org/ZDB-ALT-250528-1).

### Plasmid construction and reagents

The cDNA fragments encoding the P protein (DQ916055.1), N protein (MW020573.1), and G proteins (EU370915.1) were amplified by RT-PCR from SVCV-infected cell RNA. The amplified genes were then subcloned into the pCMV-Myc (Clontech), pCMV-HA (Clontech), or pCMV-Flag (Clontech) vectors, respectively. All constructs were verified by DNA sequencing. Poly(I:C) was purchased from InvivoGen (San Diego, CA) and transfected using Neofect DNA transfection reagent at a final concentration of 1 mg/mL. Sodium L-Lactate was purchased from Sigma-Aldrich. The antibodies used were as follows: anti-Myc antibody (Santa Cruz Biotechnology), anti-Flag antibody (Sigma-Aldrich), anti-HA antibody (Covance), anti-bactin antibody (Abclonal), anti-svcv P (AtaGenix) antibody, and anti-L-Lactyl (PTM bio) antibody.

### Virus infection and cytopathic effect assay

The spring viremia of carp virus (virus strain OMG067) was propagated in EPC cells until CPE was observed. The cell culture medium was then harvested and cryopreserved at –80°C. Viral titers were determined using a 50% tissue culture-infective dose (TCID_50_) assay on EPC cells.

To infect zebrafish larvae with SVCV, thirty 4-dpf larvae were immersed in 4 mL of egg water supplemented with 1 mL of viral suspension (~2.5 × 10^7^ TCID_50_/mL) in 60 mm cell culture dishes. The infection proceeded for 24 h at 28°C, with triplicate experimental groups. Post-infection RNA extraction was performed using TRIzol reagent, followed by qRT-PCR analysis of viral load.

To infect adult zebrafish with SVCV, 3-month-old zebrafish received i.p. injections of a 10 μL SVCV inoculum (~2.5 × 10^7^ TCID_50_/mL), while the control groups received equivalent volumes of sterile cell culture medium. Forty-eight hours post-injection, the zebrafish were anesthetized with tricaine methanesulfonate and dissected. Their brains, hearts, livers, spleens, and kidneys were collected and stored at −80°C for further qRT-PCR assays.

To evaluate the antiviral effect of sodium lactate (NaLac), EPC cells were pretreated with NaLac at a working concentration of 5 mM for 24 h, after which they were challenged with SVCV at a specified multiplicity of infection (MOI). Following a 48 h incubation period, the cells were fixed with 4% paraformaldehyde (PFA) and stained with 1% crystal violet to visualize CPE. For virus titration, the culture medium was collected at 48 h post-infection and used for a plaque assay.

### Sodium lactate and lactic acid treatment assay

For pre-treating zebrafish larvae with sodium lactate, the larvae were immersed in water at a final concentration of 5 mM for over 12 h prior to infection. For the pre-treatment of adult zebrafish with sodium lactate, the fish were fed a specially formulated diet supplemented with sodium lactate for over 30 days, after which the subsequent experiments were carried out. The diet consists of fish meal (21.36%), soybean protein concentrate (38.83%), carboxymethyl cellulose (4.86%), starch (19.42%), fish oil (7.77%), a vitamin premix (1.94%), a mineral premix (2.91%), and sodium lactate (2.91%). The feed ingredients were crushed and sieved through 80 mesh, then added in proportion and mixed. The feed is produced in the form of precipitated hard pellets using a ring mode hard pellet unit, then air-dried and slightly crushed using a small pulverizer to leave a particle size of over 40 mesh but not over 60 mesh. The control group was fed a diet devoid of sodium lactate, while the diets of the experimental and control groups contained the same proportions of ingredients, except for sodium lactate.

### Quantitative real-time PCR analysis

Total RNA was extracted from cells, embryos (*n* = 30), and tissues (*n* = 3) using Transzol UP (*TRANS*, Cat#ET111-01) according to the manufacturer’s protocol. cDNAs were synthesized using the EasyScript One-Step gDNA Removal and cDNA Synthesis SuperMix (*TRANS*, Cat#AE311-03). PerfectStart Green qPCR Super (*TRANS*, Cat#AQ601-02) was used for quantitative RT-PCR assays. qRT-PCR was performed with three biological replicates, and each experiment was repeated at least three times independently. In ZFL and EPC cells, β-actin was used as the internal control. In HEK293T and THP-1 cells, GAPDH was used as the internal control. The primers used in the qRT-PCR assays are listed in Table S1.

### Hematoxylin and eosin staining

Liver tissue was harvested from both control and virus-infected zebrafish and fixed overnight in 4% PFA at room temperature. The samples were then embedded in paraffin, sectioned into thin slices, and stained with hematoxylin and eosin for histological analysis. Light microscopy was used to examine the liver tissues for morphological alterations.

### Co-immunoprecipitation assay and western blot

Anti-Flag conjugated agarose beads were obtained from YEASEN. Western blotting and co-immunoprecipitation assays for ectopically expressed proteins were performed as previously described. Blot imaging was conducted using the Fuji Film LAS4000 mini luminescent imaging system. Protein quantification was then achieved through band density analysis using Multi Gauge V3.0 software.

### Immunofluorescence confocal microscopy

The cultured cells were adhered to glass-bottomed Petri dishes, fixed with 4% PFA for 20 min at room temperature, and then blocked with 1% bovine serum albumin supplemented phosphate-buffered saline (PBS) for 1 h.

The immunofluorescence staining protocol comprised sequential incubations: primary antibody probing at 4°C overnight, followed by species-matched, fluorophore-conjugated secondary antibodies at room temperature for 1 h, protected from light. Nuclear counterstaining was achieved using 1 μg /mL DAPI (Biosharp, Cat# BL105A) in PBS for 5 min. High-resolution confocal imaging was performed using a Leica laser-scanning confocal microscope.

### Identification of SVCV lactylation site(s) by mass spectrometry

HEK293T cells were transfected with Flag-SVCV-N or Flag-SVCV-P plasmid. The cell lysate was then immunoprecipitated with anti-Flag antibody-conjugated agarose beads overnight. The immunoprecipitated SVCV-N and SVCV-P proteins were separated by 8% SDS-PAGE, the resulting bands were excised from the gel and analyzed by mass spectrometry at Protein Gene Biotech in Wuhan, Hubei, China.

### Statistical analysis

Statistical analysis was performed using GraphPad Prism version 8.0 (unpaired *t*-tests). The data are presented as the mean ± SEM of three technical replicates. Each experiment was independently repeated at least three times. For the zebrafish survival analysis, graphs were generated using the Kaplan–Meier method, and the survival curves were analyzed using log-rank analysis. A *P* value of less than 0.05 was considered significant. Statistical significance is represented as follows: *P < 0.05, **P < 0.01, ***P < 0.001, ****P < 0.0001.

## SUPPLEMENTAL MATERIAL

Supplemental material spectrum.02473-25-s0001.docx Table S1; Fig. S1 to S4.

ASM does not own the copyrights to Supplemental Material that may be linked to, or accessed through, an article. The authors have granted ASM a non-exclusive, world-wide license to publish the Supplemental Material files. Please contact the corresponding author directly for reuse.
