# Supplementary material for: Lactylation of its phosphoprotein interferes with the replication of the spring viremia of carp virus
Source: Microbiol Spectr. 2026 Jul 6;14(8):e02473-25. doi: 10.1128/spectrum.02473-25 (PMC13436216; doi:10.1128/spectrum.02473-25)
Supplement: Supplementary file 1 [file spectrum.02473-25-s0001.docx]

**Supplemental Table 1.**

**The primer sequences**

| *Primer* | Sequence (5'-3') |
| --- | --- |
| ZF-*ldha*-RT | CCTTCTCAAGGATCTGACCG |
|  | ACACTGTAATCTTTATCCGC |
| ZF-*β-actin*-RT | AGGTCCAATGTGATACCGC |
|  | GCGCCATACAGAGCAGAA |
| ZF-*ifn1*-RT | GAGCACATGAACTCGGTGAA |
|  | TGCGTATCTTGCCACACATT |
| ZF-*lta*-RT | AAGCCAAACGAAGGTCA |
|  | AAGCCAAACGAAGGTCA |
| ZF-*mxc*-RT | GAGGCTTCACTTGGCAACTC |
|  | TTGTTCCAATAAGGCCAAGC |
| ZF-*tnfα*-RT | GCTGGATCTTCAAAGTCGGGTGTA |
|  | TGTGAGTCTCAGCACACTTCCATC |
| ZF-*il1β*-RT | TGGACTTCGCAGCACAAAATG |
|  | GTTCACTTCACGCTCTTGGATG |
| ZF-*il6*-RT | CCGAAATATCTGGAGACGAAGT |
|  | GGTCTGAAGGTTTGAGGAGAG |
| SVCV-M-protein-RT | TACTCCTCCCACTTACGA |
|  | CAAGAGTCCGAGAAGGTC |
| SVCV-N-protein-RT | TGAGGTGAGTGCTGAGGATG |
|  | CCATCAGCAAAGTCCGGTAT |
| SVCV-G-protein-RT | CGACCTGGATTAGACTT |
|  | AATGTTCCGTTTCTCACT |
| SVCV-L-protein-RT | GCCCACTTTGCATCCAGTCC |
|  | GAGATGCCACAGACTCCTCC |
| SVCV-P-protein-RT | TTGGACCTGGGATAGTGA |
|  | CTTGCTTGGTTTGTGGG |
| EPC-*β-actin*-RT | CACTGTGCCCATCTACGAG |
|  | CCATCTCCTGCTCGAAGTC |
| EPC-*ifn*-RT | ATGAAAACTCAAATGTGGACGTA |
|  | GATAGTTTCCACCCATTTCCTTAA |
| EPC-*isg15*-RT | CAGCCTTGAGGATGATTCCAG |
|  | TGCCGTTGTAAATCAGTCG |
| EPC-*viperin*-RT | AGCGAGGCTTACGACTTCTG |
|  | GCACCAACTCTCCCAGAAAA |
| Human-*GAPDH*-RT | GACAAGCTTCCCGTTCTCAG |
|  | GAGTCAACGGATTTGGTGGT |
| Human-*IFNB1*-RT | TTGTTGAGAACCTCCTGGCT |
|  | TGACTATGGTCCAGGCACAG |
| Human-*ISG15*-RT | GAGAGGCAGCGAACTCATCTT |
|  | CCAGCATCTTCACCGTCAGG |
| Human-*CXCL10*-RT | GCTATACTGAGGTGCTATGTTC |
|  | GGAGGATGGCAGTGGAAGTC |

**FIG. S1.**

**Supplemental FIG. 1. Sodium lactate treatment does not affect the stability of SVCV’s structural proteins.**

(A-C) Western blot analysis showed no change in the protein levels of the SVCV nucleoprotein (N) (A), phosphoprotein (P) (B), and glycoprotein (G) (C) following sodium lactate treatment. HEK293T cells were transfected with 2 μg of each plasmid construct and then treated with 5mM sodium lactate.

**FIG. S2.**

**Supplemental FIG. 2. Effect of lactate and sodium lactate on the RLR signaling pathway in mammalian cells following VSV infection or poly (I:C) treatment.**

(A-C) In THP-1 cells, treatment with either lactate or sodium lactate (5 mM) significantly suppressed the VSV-induced expression of the typical antiviral genes, *IFNB1* (A), *CXCL10* (B), and *ISG15* (C). The cells were pretreated with lactate or sodium lactate (5 mM) for 12 h prior to viral stimulation. (D-F) Lactate treatment inhibited the expression of typical antiviral genes, *IFNB1* (D) , *CXCL10* (E), and *ISG15* (F) induced by poly (I:C) in HEK293T cells.

The data presented are the means ± SEM, based on one representative experiment performed in triplicate. *p < 0.05; **p < 0.01; ***p < 0.001; ****p < 0.0001.

**FIG. S3.**

**Supplemental FIG. 3**. **Effect of sodium lactate on the RLR signaling pathway in fish cells following poly (I:C) treatment.**

(A and B) In EPC cells, sodium lactate treatment did not suppress the expression of *ifn* (A) and *viperin* (B) induced by poly(I:C). The cells were pretreated with lactate or sodium lactate (5 mM) for 12 h prior to the addition of poly (I:C).

**FIG. S4**

**Supplemental FIG. 4.** **Lactylated lysine residues in the SVCV-N and P proteins are identified by mass spectrometry.**

(A) Lactylation residues in SVCV-N and P proteins. HEK293T cells were transfected with Flag-SVCV-N or Flag-SVCV-P for 24 h and then subjected to mass spectrometry analysis.(B-C) The lactylated residues in SVCV-N identified by mass spectrometry analysis. HEK293T cells transfected with Flag-SVCV-N for 24 h were analyzed by mass spectrometry. (D-F) The lactylated residue in SVCV-P identified by mass spectrometry analysis. HEK293T cells were transfected with Flag-SVCV-P for 24 h and then subjected to mass spectrometry analysis.
